# Human papilloma virus vaccination programs reduce health inequity in most scenarios: a simulation study

**DOI:** 10.1186/1471-2458-12-935

**Published:** 2012-10-31

**Authors:** Natasha S Crowcroft, Jemila S Hamid, Shelley L Deeks, John Frank

**Affiliations:** 1 Infectious Diseases, Public Health Ontario, 480 University Avenue, Suite 300, Toronto, Ontario, M5G 1V2, Canada; 2 Laboratory Medicine and Pathobiology, University of Toronto, 1 King's College Circle, Toronto, Ontario, M5S 1A8, Canada; 3 Dalla Lana School of Public Health, University of Toronto, 155 College Street, Health Science Building, 6th floor, Toronto, Ontario, M5T 3M7, Canada; 4 Clinical Epidemiology and Biostatistics, McMaster University, 1280 Main Street West, Hamilton, Ontario, L8S 4L8, Canada; 5 Pathology and Molecular Medicine, McMaster University, 1280 Main Street West, Hamilton, Ontario, L8S 4L8, Canada; 6 Scottish Collaboration for Public Health Research and Policy, Edinburgh, Ontario, Canada; 7 University of Edinburgh, Old College, South Bridge, Edinburgh, Scotland, EH8 9YL, UK

## Abstract

**Background:**

The global and within-country epidemiology of cervical cancer exemplifies health inequity. Public health programs may reduce absolute risk but increase inequity; inequity may be further compounded by screening programs. In this context, we aimed to explore what the impact of human papillomavirus (HPV) vaccine might have on health equity allowing for uncertainty surrounding the long-term effect of HPV vaccination programs.

**Methods:**

A simple static multi-way sensitivity analysis was carried out to compare the relative risk, comparing after to before implementation of a vaccination program, of infections which would cause invasive cervical cancer if neither prevented nor detected, using plausible ranges of vaccine effectiveness, vaccination coverage, screening sensitivity, screening uptake and changes in uptake.

**Results:**

We considered a total number of 3,793,902 scenarios. In 63.9% of scenarios considered, vaccination would lead to a better outcome for a population or subgroup with that combination of parameters. Regardless of vaccine effectiveness and coverage, most simulations led to lower rates of disease.

**Conclusions:**

If vaccination coverage and screening uptake are high, then communities are always better off with a vaccination program. The findings highlight the importance of achieving and maintaining high immunization coverage and screening uptake in high risk groups in the interest of health equity.

## Background

Cervical cancer exemplifies health inequity, both within nations and globally [1]. Inequities have persisted even within countries with good screening programs; there is hope that primary prevention through vaccination may address this [2]. A substantial body of work has indicated significant benefits and cost-benefits of vaccination against the infectious cause of cervical cancer, human papilloma virus (HPV) [3,4]. Two vaccines are available to protect against genotypes responsible for about 70% of cervical cancers [5]. These vaccines have been found to be very efficacious and safe and programs have been implemented in various countries throughout the world, including Canada, Australia, the United Kingdom and the United States. HPV vaccination programmes have nevertheless aroused some controversy. The vaccine is expensive, raising questions about alternative public health gains in countries with existing cervical cancer screening programs, which could have been obtained for the same investment. Non-substantiated fears have been raised by some, about the impact of the vaccine on sexual behaviour; parents who believe the vaccine might have a negative influence on sexual behaviour are less likely to intend to vaccinate daughters [6]. Other commentators [7] have pointed out that this vaccine is unique, in that it could potentially lead to the decreased utilization of the cervical screening program, already established as effective, by women unclear about the vaccine’s incomplete coverage of oncogenic viral genotypes, [8,9], thereby paradoxically increasing the future burden of invasive cervical cancer from the much less common non-vaccine strains. It is important to note that cervical screening is not primary prevention since it provides early detection of cervical abnormalities; it is only effective among women who participate in the program and there are well documented social inequities regarding program participation [10-12].

There are a number of uncertainties, inevitable with any new vaccine, which require careful monitoring of the “natural experiment” currently taking place in jurisdictions such as Canada, where different schedules for publicly funded HPV vaccination programs have been implemented at different times and for age groups ranging from grade 4 to 8 (ages 9–13 years) [13]. The programs are all being delivered to girls by public health through schools, however the school grade targeted varies throughout the country. Surveillance of all aspects of new vaccination programmes - adverse events, coverage, attitudes and disease incidence – is essential. One of several reasons for this is that vaccination programmes may have paradoxical effects in increasing burden of disease [14] and inequity in health [15]. However, as the vaccine is delivered through school-based programs, and children in Canada are legally required to attend schools, inequity may be diminished. In the context of uncertainty about key parameters such as duration of vaccine protection, and the impact of the programme on behaviour related to screening uptake, the question arises whether there are realistic scenarios in which HPV vaccination programmes could cause relative or absolute harm to particular groups in society.

A root cause of inequity in cervical cancer is poverty, mediated biologically by increased risk of sexual exposure to HPV and reduced detection, appropriate follow-up and treatment for preclinical abnormalities. Cervical cancer is also a disease of poverty via psycho-social mechanisms, such as a lack of power for women around sexual relations (in many societies, but generally associated particularly with lower levels of female education) and lack of understanding of the disease [16].

Although current vaccines protect against two of the HPV types which cause around 70% of cervical cancers, another 13 types are established high-risk and a further 3 are probably high-risk [5]. Because of these other types, as well as the fact that no vaccine is 100% effective, cervical cancer screening programmes need to continue in some form, regardless of vaccination programmes [5,17]. Implementation of such “competing” programmes (in the sense that they may compete for the attention and compliance of women at risk) may harm some groups if, post-vaccination, behavioural changes either increase the risk of acquisition of non-vaccine oncogenic HPV types, or reduce the uptake of effective screening and follow-up treatment of pre-clinical abnormalities. An additional possible issue would be that as more advanced lesions become rarer, high-volume Pap-smear cytologists may become less adept at recognizing them. These perverse effects may be potentiated if the extent and/or duration of vaccine effectiveness is lower than anticipated (data on the long-term duration of protection are currently accumulating and are approximately five years ahead of vaccination programs). Beneficial effects of vaccination may, on the other hand, be potentiated, if the vaccine confers significant indirect (“herd”) protection by reducing the circulation of the HPV vaccine strains or if it confers protection for women who would not have participated in the screening program. The overall impact of a vaccination programme on the risk of cervical cancer thus depends on the interplay of several factors. We aimed to find out whether it is possible using a simple approach to discover plausible scenarios in which corresponding population subgroups could be at increased risk of invasive cervical cancer following the implementation of an HPV vaccination programme, thereby potentially increasing inequity in the whole population.

## Methods

We carried out a multi-way sensitivity analysis using a simple static mathematical model developed to assess the impact of different prevention strategies on cervical cancer relative to the state before a vaccination program was implemented. The simulation generates a number of scenarios representing a set of conditions that might apply to a particular subgroup of the population. The terms scenario and subgroup are therefore used interchangeably below.

We considered each of many possible subgroups of the population, defined by specific values of several variables that co-determine invasive cervical carcinoma risk, and estimated whether each subgroup would be better or worse off with a vaccination programme in place, given the presence of cervical screening. The parameters were varied within a wide range that included plausible values to allow for different characteristics of risk groups in any population (Table 1). The possible impact of HPV vaccination programmes for teenage girls on the prevalence of circulating vaccine strains through herd immunity is currently unknown. We estimated indirect effects at exposure reductions of 60%, 40% and 20%. Vaccine effectiveness was considered between 50% and 90%. The lower end of the range for the vaccine effectiveness value was set at 50% to allow for possible waning of immunity during mid-adult life, to capture longer-term effectiveness, as opposed to efficacy, of the vaccine. Mid-adult life is a time when sexual activity for many women still leads to HPV exposure, with increasing rates of breakdown of marital relationships and new sexual partner initiation in later years [18], and given that many years will have elapsed since they were vaccinated by the pre-pubertal programs now in place. In published dynamic models, vaccine efficacy has been estimated at 90-100% [19-21]. The duration of immunity is unknown and will likely depend in part on frequency of ongoing exposure to HPV and whether this provides immunological boosting. For other viral vaccines such as measles, vaccine-derived immunity has been shown to decline more rapidly than natural immunity, at least in part due to the success of programs in eliminating viral circulation and boosting. Cross protection against other genotypes was not included in the model. We investigated coverage in the range of 20%-90% to account for high coverage in some developed countries and low coverage in some developing countries (this may be true for some religious and other subgroups as well). Baseline screening sensitivity was previously estimated to be 59% [10] - we, therefore, focus on this level of baseline sensitivity when presenting the results of our simulation study, although a similar range of estimates as used for vaccine effectiveness was also considered. Lifetime screening participation of between 10% and 90% was investigated.

*R:*: Risk of infections which would cause invasive cervical cancer if neither prevented nor detected;

*V:*: Vaccine effectiveness;

*C:*: Vaccination coverage;

*S:*: Screening uptake prior to the introduction of the program;

*E:*: Baseline lifetime screening sensitivity (the sensitivity of the screening program);

*δPv:*: Proportional change in risk (*R*) caused by vaccine strains if neither prevented nor detected, occurring as a result of herd immunity or behavioural change;

*δPu:*: Proportional change in risk (*R*) caused by non-vaccine strains if neither prevented nor detected, occurring as a result of behavioural change or strain replacement;

*δS:*: Proportional change in screening uptake;

*δE:*: Proportional change in lifetime screening sensitivity;

*R*_*1:*_: Risk of undetected infections which would cause invasive cervical cancer for a woman living in a population without a vaccination programme;

*R*_*2:*_: Risk of undetected infections which would cause invasive cervical cancer for a woman living in a population with a programme.

**Table 1:** Ranges of parameters considered in the simulation study

**V**	**C**	**S**	**E**	**δPv**	**δPu**	**δS**	**δE**
90%	90%	90%	90%	−0.6	−0.6	−0.6	−0.6
80%	80%	80%	80%	−0.4	−0.4	−0.4	−0.4
70%	70%	70%	70%	−0.2	−0.2	−0.2	−0.2
60%	60%	60%	60%	0	0	0	0
50%	50%	50%	50%	0.2	0.2	0.2	0.2
	40%	40%		0.4	0.4	0.4	0.4
	30%	30%		0.6	0.6	0.6	0.6
	20%	20%					
		10%					

V =Vaccine effectiveness.

C = Vaccination coverage.

S = Screening uptake.

E = Baseline lifetime screening sensitivity (the sensitivity of the screening program).

δPv = Proportional change in the risk of infections caused by vaccine strains which would cause cervical cancer if nether prevented nor detected.

δPu = Proportional change in the risk of infections caused by non-vaccine strains which would cause cervical cancer if nether prevented nor detected.

δS =Proportional change in screening uptake.

δE = Proportional change in lifetime screening sensitivity.

For a woman living in population without a vaccination programme, risk of undetected infections which would cause invasive cervical cancer can be estimated as

(1) $$R_{1}=R*\left(1-S*E\right)$$

However, for a woman under the vaccination program, the risk of getting these life threatening infections can be described as

(2) $$R*\left(1+\delta P\upsilon +\delta Pu\right)\text{,}$$

Where, *δPv* and *δPu* are changes (increases or decreases) attributed to vaccine or non-vaccine strain exposures.

Risk reduction due to vaccination is estimated as,

(3) $$0.7*R*C*V+\delta P\upsilon *RC*V=R*C*V*\left(0.7+\delta P\upsilon \right)\text{,}$$

Where 0.7 accounts for the protection obtained from currently available vaccines against two of the HPV types which cause approximately 70% of cervical cancers.

Therefore, the risk of undetected infections can now be given as

(4) $$\begin{array}{l} \left(R*\left(1+\delta P\upsilon +\delta Pu\right)-R*C*V*\left(0.7+\delta P\upsilon \right)\right)\\ \;=R*\left(\left(1+\delta P\upsilon +\delta Pu\right)-\left(C*V*\left(0.7+\delta P\upsilon \right)\right)\right) \end{array}$$

Similarly, changes in uptake of screening and of the effectiveness of screening can be incorporated in the estimates, consequently, *R*_*2*_ becomes

(5) $$R_{2}=R*\left(\left(2+\delta P\upsilon +\delta Pu\right)-C*V*\left(0.7+\delta P\upsilon \right)*\left(1-(S*E*\left(1+\delta S\right)\left(1+\delta E\right)\right)\right)$$

The relative risk can then be given as

(6) $$\begin{array}{l} RR=\frac{R_{2}}{R_{1}}\\ \;=\frac{\left(\left(1+\delta P\upsilon +\delta Pu\right)-C*V*\left(0.7+\delta P\upsilon \right)\right)*\left(1-\left(S*E*\left(1+\delta S\right)\right)\right)}{1-S*E} \end{array}$$

If the relative risk (RR) is less than 1 then the outcome of the vaccination programme was considered to be better and if greater than or equal to 1, then the outcome of vaccination was worse than the previous state. Vaccine adverse events were not included in the analysis, and the vaccine is relatively expensive, so that *R*_*1*_*=R*_*2*_ with a vaccination programme was not considered a satisfactory outcome. On the other hand, early indications are that the vaccine is safe and well tolerated with the only excess adverse event reported so far being anaphylaxis, with a number needed to harm (NNH) of around 40,000 per dose [22]. Similarly, adverse effects of screening were also not included in the analysis.

Our primary outcome of interest is the percentage of scenarios/subgroups, of the all possible combinations of parameter values which are considered, in which *RR* ≥ 1. The actual percentage is less important than the trends across different levels of parameters and the range of scenarios in which the outcome is not better with a vaccination program. We are interested in scenarios in which subgroups may be worse off as an indicator of the extent to which vaccination programs may result in inequity. These scenarios also represent the situations in which certain characteristics and circumstances may cluster to cause disadvantage to population subgroups and hence create inequity.

Applying the range of parameters considered in the simulation (Table 1), the number of possible combinations is 8*9*5*7*7*7*7= 864,360 for each level of vaccine effectiveness, giving a total of 4,321,800 scenarios. However, we removed 527, 898 scenarios that led to zero or negative risk as being practically irrelevant although they are mathematically valid. This occurs when (1 + *δS*) * (1 + *δE*) ≥ 1/(*E* * *S*), and one such example is when baseline screening uptake and sensitivity are set to 70% and 80% , respectively while change in uptake and sensitivity are allowed to be 0.6. This leads to scenarios which are unrealistic, which are thus removed from our analysis/investigation. Therefore, the total number of subgroups/scenarios investigated in our paper 3,793,902. In addition we carried out sensitivity analyses to see the impact of two variables at a time in different scenarios. The simulation and data analysis is performed in the R statistical package [23].

## Results

**Figure 1:**
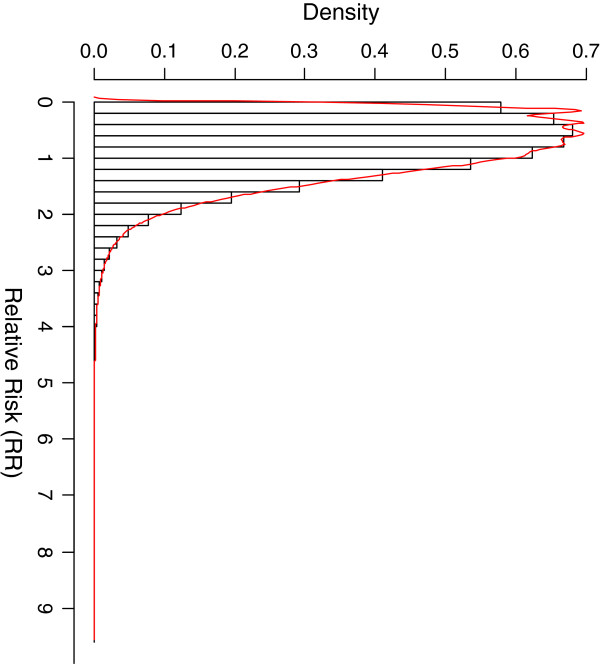
**The distribution of RR when all the eight parameters considered are allowed to vary within the range provided in Table**1.

**Figure 2:**
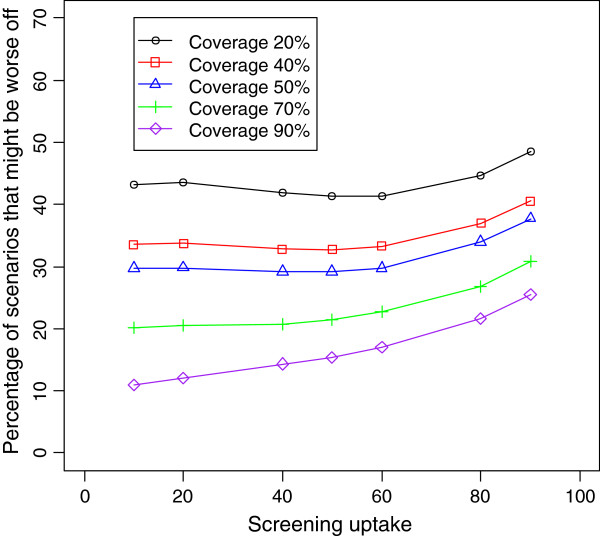
**Percentage of scenarios in which a subgroup might be worse off, for different levels of coverage and baseline screening uptake.** Vaccine effectiveness is set at 90% and baseline sensitivity is set at 60%. Other variables are allowed to vary within the range provided in Table 1.

**Figure 3:**
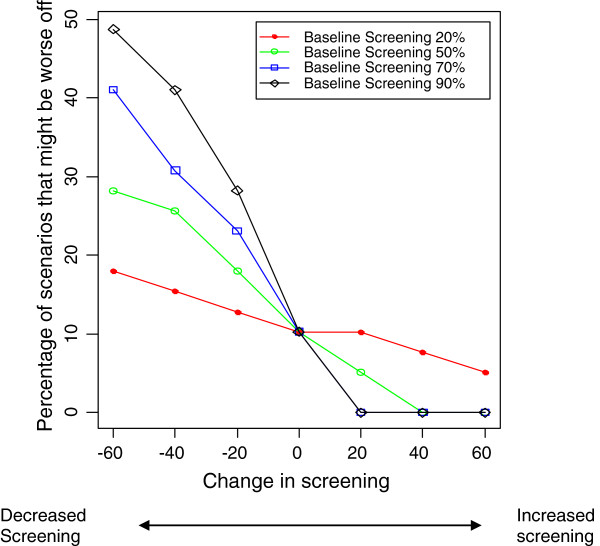
**Percentage of scenarios in which a subgroup might be worse off, at different levels of change in screening uptake.** Vaccine effectiveness and coverage are set at 90%, and screening sensitivity is maintained at 60%.

**Figure 4:**
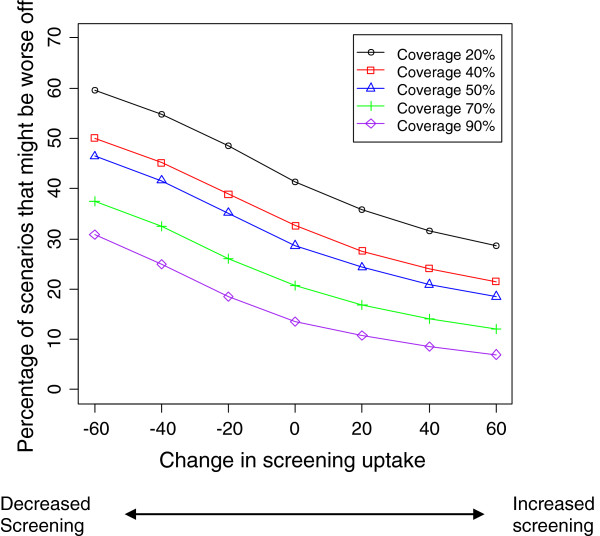
**Percentage of scenarios in which a subgroup might be worse off in a scenario where baseline screening is high, according to coverage and different levels of change in screening uptake.** V=90%, E=60%, S=80%, the rest of the variables are assumed to vary according to Table 1.

**Figure 5:**
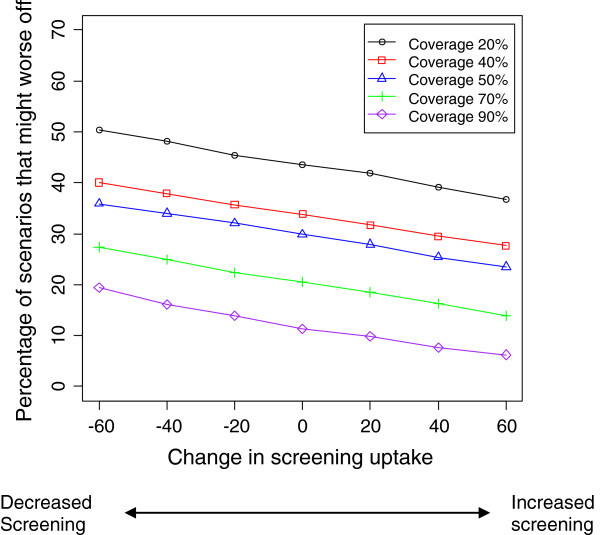
**Percentage of scenarios in which a subgroup might be worse off in a scenario where baseline screening is high, according to coverage and different levels of change in screening uptake.** V=90%, E=60%, S=20%, the rest of the variables are assumed to vary according to Table 1.

The results from our simulation study show that the outcome of vaccination would be better in the majority of scenarios/subgroups (RR<1 in 63.9% of scenarios considered) but worse in a substantial proportion of scenarios considered (RR ≥1 in 36.1% of the scenarios). The distribution of RR is heavily skewed to the left (the direction of a beneficial effect), where RR < 1.2 for more than 75% of the scenarios considered, RR > 2 for less than 5% of the scenarios and less than 1% of the scenarios result in RR >3 (Figure 1). This indicates that in most scenarios, the population subgroup benefits from the vaccination programme. This is consistently observed in our simulation regardless of vaccine effectiveness and coverage although the actual percentage values vary with each (Table 2, Figures 2, 3, 4 and 5).

**Table 2:** Distribution of relative risk (RR) at different levels of vaccine effectiveness (V) and coverage (C)

**V**	**C**	**Percentage of Scenarios**				
		**RR < 1**	**RR < 1.2**	**RR > 1**	**RR > 2**	**RR > 3**
90	20	54.92	66.21	45.08	7.70	1.52
	50	66.94	77.60	33.06	3.59	0.71
	70	74.92	84.89	25.08	2.05	0.39
	90	81.25	90.58	18.75	1.25	0.22
70	20	53.14	64.46	46.86	8.61	1.71
	50	63.01	73.79	36.99	4.73	0.95
	70	69.05	79.44	30.95	3.19	0.62
	90	74.92	84.89	25.08	2.05	0.39
50	20	51.44	62.78	48.56	9.48	1.91
	50	58.28	69.42	41.72	6.3	1.25
	70	63.01	73.79	36.99	4.73	0.95
	90	66.94	77.60	33.06	3.59	0.71

Footnote: If RR>1, the outcome is worse in that scenario/subgroup.

As expected, the benefit of the vaccine programme increases as vaccine effectiveness and coverage increase, indicating that achieving and maintaining high coverage is important in all subgroups to get a maximal benefit from the vaccine program. This is true regardless of vaccine effectiveness. When vaccine effectiveness is set at 50% (well below current estimates), while other population parameters are allowed to vary, 59.3% of the scenarios still result in a beneficial outcome with RR < 1. The distribution remains heavily skewed to the left. Only 6% of the populations had RR > 2 and RR > 3 for about 1% of the scenarios. Similarly, for vaccine effectiveness of 90%, 68.4% of the populations result in RR < 1 and RR > 1 was obtained for 31.6% of the population, RR < 1.2 for 79% of the populations, RR > 2 for less than 3% of the populations and less than 1% of the scenarios result in RR > 3.

When vaccine effectiveness is set at 90% and coverage is very low (20%), the percentage of scenarios in which the population might be worse off with a vaccination programme is quite high (45%) although the relative risk is still less than 1.2 for 66% the scenarios. However, when coverage is increased to 70%, the percentage of scenarios that are worse off decreases substantially (25.1%). For 90% coverage, the percentage of scenarios in which populations could potentially be worse off is only 18.8%, and the percentage of scenarios with RR < 1.2 increased to 90.6%. Table 2 shows the distribution of RR for different values of vaccine effectiveness and coverage. Figures 2, 3, 4 and 5 show the impact of varying coverage and screening uptake or changes in screening uptake and reinforce the baseline screening level and coverage to be the most important factors to consider. Note that the effect of changes in screening uptake or sensitivity would be identical because our formula is symmetrical.

The proportion of scenarios in which RR<1 was lower if screening uptake was higher at baseline and then declined (Table 3, Figures 2, 3). If screening participation is maintained at baseline levels, whatever they may be, and vaccine coverage is high, the outcome is good in all scenarios (Table 3).

**Table 3:** Percentage of scenarios in which a subgroup might be worse off at different levels of baseline screening uptake

**S**	**δS**	**Percentage of Scenarios**			
		**E = 60%**		**E = 90%**	
		**δE≠0**	**δE=0**	**δE≠0**	**δE=0**
20	−0.6	19.41	17.95	23.08	23.08
	−0.4	16.12	15.38	19.05	17.97
	−0.2	13.92	12.82	15.75	12.82
	0	11.36	10.27	10.99	10.26
	0.2	9.89	10.26	9.52	7.69
	0.4	7.69	7.69	6.96	5.13
	0.6	6.28	5.13	5.49	0
50	−0.6	29.67	28.21	41.39	43.59
	−0.4	24.54	25.64	32.60	33.33
	−0.2	17.95	17.95	22.71	25.64
	0	12.45	10.26	16.11	10.26
	0.2	9.89	5.13	11.72	0
	0.4	7.33	0	10.25	0
	0.6	5.86	0	7.91	0
70	−0.6	38.83	41.03	56.41	58.97
	−0.4	30.77	30.77	44.69	48.72
	−0.2	21.61	23.08	30.77	35.90
	0	15.02	10.26	24.59	10.26
	0.2	10.98	0	20.46	0
	0.4	8.42	0	16.67	0
	0.6	6.97	0	15.92	0
90	−0.6	49.08	48.72	75.46	76.92
	−0.4	37.73	41.03	62.27	66.67
	−0.2	26.01	28.21	47.95	53.85
	0	18.32	10.26	37.67	10.26
	0.2	15.16	0	33.87	0
	0.4	12.56	0	30.57	0
	0.6	11.83	0	32.03	0

S = Screening uptake.

E = Baseline screening sensitivity (the sensitivity of the test).

δS =Proportional change in screening uptake.

δE = Proportional change in screening sensitivity.

Coverage and vaccine effectiveness are set at 90%, baseline sensitivity is set at 60% and 90%.

The greater the increase in non-vaccine strains, the greater the proportion of scenarios with a worse outcome, reaching 30% of scenarios if non-vaccine strains increased by 40%. However, if non-vaccine strains increased by only 20%, the population is better off in 90% of scenarios. Our simulation results indicate that the percentage of scenarios that are worse off can be reduced further if coverage, screening uptake and sensitivity are maintained or increased (Figures 2, 3 4 and 5). While baseline screening uptake, sensitivity and the risk of infection prior to introduction cannot be changed and vaccine effectiveness is also a fixed characteristic of the program, changes in screening uptake after introduction and vaccination coverage are amenable to intervention so the finding that these are also the critical elements in a sensitivity analysis is important.

Proportional improvements in cytological screening effectiveness reduced the number of scenarios in which sub-populations might be worse off with a programme (data not shown). The effect was more modest in the different baseline screening scenarios when vaccine effectiveness was set at 90%, but for every level of screening effectiveness the proportion of scenarios in which the outcome was worse, after vaccination, remained very small as long as coverage is high and screening uptake is maintained.

## Discussion

This analysis is very simplistic and aims as much to raise the issue of consequences, for different groups in society, as to yield precise estimates of program effectiveness. It should be regarded as an exploratory sensitivity analysis and the basis for further work. More sophisticated approaches such as Monte Carlo simulations may be more methodologically pure, but perhaps less intuitive and thus more “black box” than our approach of trying out all combinations of parameter values, within what seem to be plausible ranges. We have not estimated a “prior” probability distribution for our parameters, as Bayesian methods would require, and so have remained in a state of equipoise in relation to the likelihood of these different possible future scenarios. This is a significant limitation since we know that some parameter values are much more likely than others, and that some values of different parameters which combine either to increases or decrease risk are more likely to occur in combination. Established risk factors (such as multi-partner, unprotected sexual behaviour from early in teenage life; failure to take up preventive health care - including vaccination and regular cytological screening; early smoking; and poverty) tend to cluster, as do protective factors, among various socioeconomic subpopulations, as well as certain ethnic groups in some societies. The overall effect of ignoring this non-independence of risk factor distributions in the population - i.e. clustering - is likely to make this analysis conservative with respect to the proportion of scenarios modelled in which the corresponding subgroup is substantially worse off – or much better off – after vaccination, since risk-factor clustering would tend to increase the variance of the modelled overall-risk distribution, leading to a larger number of scenarios, and corresponding fractions of most populations, with much worse-than-average and better-than-average results. In other words, this analysis may tend to underestimate the extent to which vaccination may result in inequity.

Unlike many other vaccines, even if the herd immunity effect led to an indirect impact on invasive cancer of vaccine strains, the outcome could still be poorer in some scenarios in which a subgroup of the population may find itself, because the overall impact of the program depends on other factors, including screening uptake and the prevalence of non-vaccine genotypes. But such scenarios remain in the minority. For instance, if the prevalence of circulating vaccine strains fell by 60%, then the outcome would be better for 88% of subgroups, as long as vaccination effectiveness and coverage were fixed at 90%. If the fall in vaccine strain prevalence were only 20% then the outcome would still be better in 82% of scenarios, showing that if herd immunity effects are modest this could be compensated for by declines in other parameters. Although many vaccination programmes result in herd immunity which protects everyone, vaccinated or not, they may not benefit everyone equally and, indeed, may increase inequity in health. Such iatrogenic inequity receives less attention than inequities observed in other areas of public health because the overwhelming success of vaccination programmes produces such a large absolute reduction in most individuals’ (and therefore the population-level) risk of disease. Although not addressed in the current study, adding males to the vaccination programme would have an impact on herd immunity and therefore a further benefit to women. Furthermore, the delivery of vaccine through schools, rather than primary care, may also balance the overall impact of the program in favour of increasing equity. HPV is highly transmissible, implying that to achieve significant herd immunity will require not only high effectiveness but also coverage that is significantly higher than the levels of around 50% seen jurisdictions such as Ontario [24,25].

## Conclusions

Redressing health inequity is a central theme in public health [16]. A sound conceptual framework and good information are required to quantify the impacts of all interventions’ impacts on such inequity, and to robustly evaluate any further program or policy modifications to avert particularly inequitable impacts. This simplistic analysis indicates that the population or sub-populations would be better off with a vaccination program than without one in most scenarios. In some unlikely circumstances, they may, however, be worse off with an HPV vaccination programme than without one. Ensuring that at least one person is better off while nobody is worse off after such programs are implemented (in economists’ terms, Pareto improvement) requires not only high coverage of vaccination but also good uptake of screening programmes in those groups most at risk. To ensure both requires comprehensive and linked information systems for monitoring vaccination coverage, screening registries (which ideally include information on HPV infection at a type-specific level), and cervical cancer screening uptake. This analysis suggests that to be safe and equitable, jurisdictions with vaccination programmes should aim to ensure that, in the absence of long-term data on vaccine effectiveness, coverage in all groups should be greater than 60% and that cervical screening uptake is high and sustained in all groups. The lesson from cervical screening programs is, like many screening programs, that they may increase health inequity [26]. Vaccination programmes delivered through schools may be better poised to decrease health inequities, as school attendance is mandatory and participation does not rely on attendance at an off-site health clinic. Regardless, systems need to have the capacity to identify groups at highest risk for paradoxical negative *combined* effects of screening programs and HPV vaccination, and to be linked to actions to effectively mitigate those risks. Some jurisdictions have rightly set aside resources for evaluation; those without systems in place are hoping for the best but not preparing for the worst.

## Competing interests

None of the authors has any competing interests to declare.

## Authors’ contributions

JF had the idea to do the study; NC developed the idea into a study design and carried out the initial analysis. JH advised on method and completed the statistical analysis. SD contributed further ideas for the interpretation of the data. NC drafted the paper and responses to reviewers; all authors contributed to the writing and approved final drafts.

## Pre-publication history

The pre-publication history for this paper can be accessed here:

http://www.biomedcentral.com/1471-2458/12/935/prepub
